# DNA Nanostructure-Templated Antibody Complexes Provide Insights into the Geometric Requirements of Human Complement Cascade Activation

**DOI:** 10.1021/jacs.4c02772

**Published:** 2024-05-04

**Authors:** Leoni Abendstein, Willem E. M. Noteborn, Luc S. Veenman, Douwe J. Dijkstra, Fleur S. van de Bovenkamp, Leendert A. Trouw, Thomas H. Sharp

**Affiliations:** † Department of Cell and Chemical Biology, Leiden University Medical Center, Leiden 2300 RC, The Netherlands; ‡ Department of Immunology, Leiden University Medical Center, Leiden 2333 ZA, The Netherlands; § School of Biochemistry, University of Bristol, Bristol BS8 1TD, U.K.

## Abstract

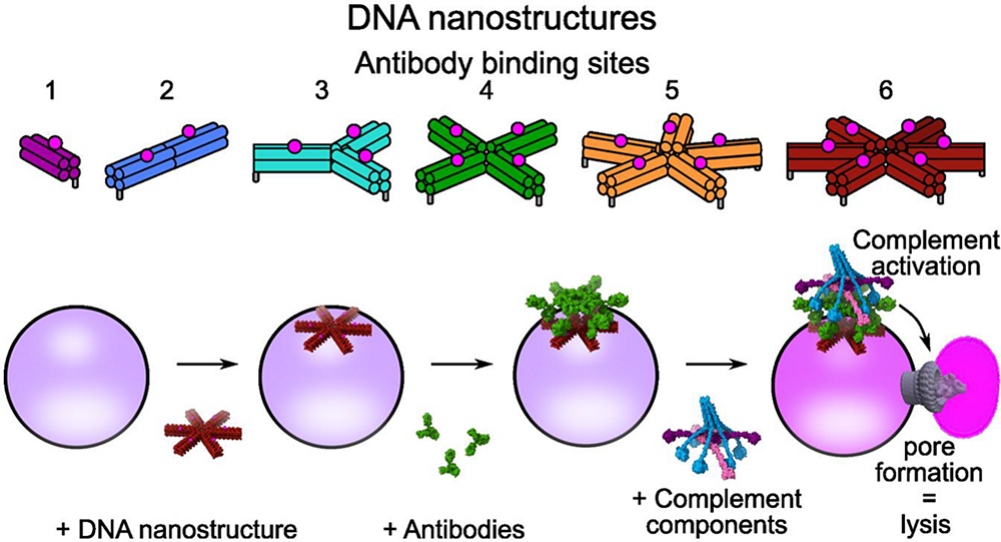

The classical complement pathway is activated by antigen-bound IgG antibodies. Monomeric IgG must oligomerize to activate complement via the hexameric C1q complex, and hexamerizing mutants of IgG appear as promising therapeutic candidates. However, structural data have shown that it is not necessary to bind all six C1q arms to initiate complement, revealing a symmetry mismatch between C1 and the hexameric IgG complex that has not been adequately explained. Here, we use DNA nanotechnology to produce specific nanostructures to template antigens and thereby spatially control IgG valency. These DNA-nanotemplated IgG complexes can activate complement on cell-mimetic lipid membranes, which enabled us to determine the effect of IgG valency on complement activation without the requirement to mutate antibodies. We investigated this using biophysical assays together with 3D cryo-electron tomography. Our data revealed the importance of interantigen distance on antibody-mediated complement activation, and that the cleavage of complement component C4 by the C1 complex is proportional to the number of ideally spaced antigens. Increased IgG valency also translated to better terminal pathway activation and membrane attack complex formation. Together, these data provide insights into how nanopatterning antigen–antibody complexes influence the activation of the C1 complex and suggest routes to modulate complement activation by antibody engineering. Furthermore, to our knowledge, this is the first time DNA nanotechnology has been used to study the activation of the complement system.

## Introduction

**Figure 1:**
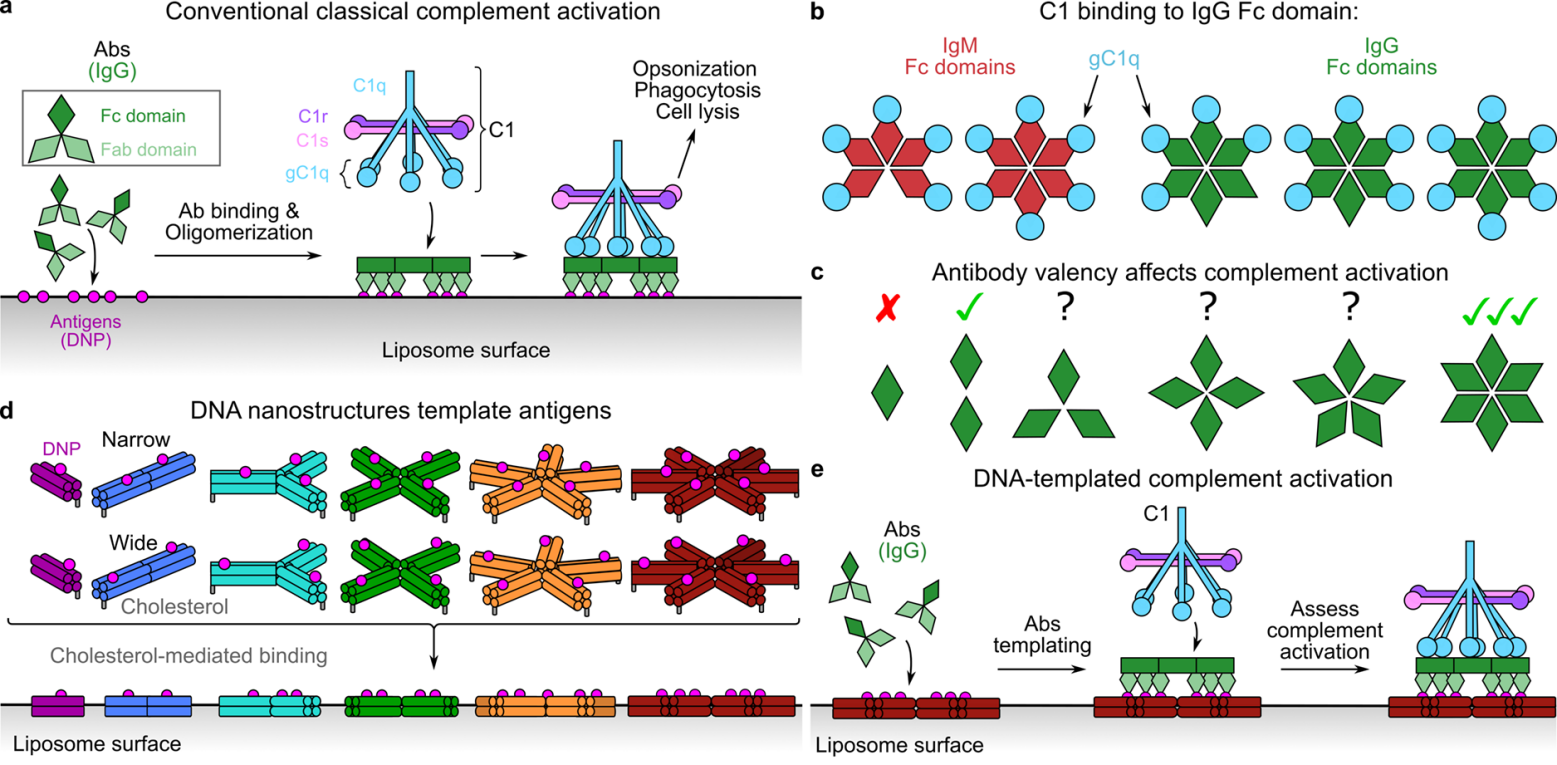
Overview of complement activation and the use of DNA nanostructures to control Ab valency. (a) Overview of classical complement initiation by the Ab-bound C1 complex. Upon binding to antigens (pink) via Fab domains (light green), the Fc domains of IgG (dark green kites) form nanopatterned platforms (dark green rectangles) that activate the C1 complex. (b) Fc domains of IgM (red kites) and IgG (green kites) oligomerize and bind to different numbers of gC1q domains (blue circles). (c) Valency is known to impact complement initiation, but the geometric requirements of how the IgG Fc monomers (green kites) affect C1 binding and activation are unknown. (d) DNA nanostructures can be used to template antigens with defined valencies and distances on lipid bilayers via cholesterol binding. (e) IgG Abs bind to DNA nanostructure-templated antigens and form platforms of defined valency to explore complement activation.

Antibodies (Abs) are important effector molecules of the human immune system. Upon binding to antigens, Abs initiate numerous immune responses, ranging from antibody-dependent cell-mediated cytotoxicity and antibody-dependent cellular phagocytosis to activation of the classical complement pathway.^1−4^ Complement forms part of the humoral innate immune response and is involved in immune defense against invading pathogens, as well as clearance of cellular debris.^4,5^ Activation of the classical pathway of complement occurs when the first complement component, the C1 complex, binds to antigen-bound Abs (Figure 1a). The C1 complex comprises C1q, which has the ability to bind Abs via the globular head domain (gC1q), and two serine proteases, C1r and C1s, that form a C1r_2_s_2_ heterotetramer (Figure 1a).^6^ Binding of C1q to Abs causes activation of C1r and C1s, which proceeds to cleave soluble C4 to form C4b, an opsonin that covalently associates with nearby molecules and membranes.^7,8^ Some C4b is bound by C2, which is then also cleaved by C1s to form the C4b2b (formerly C4b2a) complex.^9,10^ C4b2b is a C3 convertase, an enzyme complex that enables complement cascade progression by cleaving C3, resulting in coverage of membranes with multiple covalently linked opsonins, the release of anaphylatoxins, and formation of the terminal-pathway membrane attack complex (MAC), a pore that perforates the cell membrane.^4−6^

The classical pathway can be activated by antigen-bound immunoglobulin G (IgG) or IgM Abs. IgM circulates in human serum as a preformed pentamer or hexamer,^11^ but IgG exists as a monomer, and oligomerization of IgG Abs has been shown to be a requisite for complement activation. This is due to the low binding affinity of gC1q for the Ab fragment crystallizable (Fc) domains, which is in the mM range,^12−14^ resulting in two or more Abs being required for C1 binding (Figure 1b).^15−17^ More recently, it has been shown that IgG Abs oligomerize via noncovalent interaction between the Fc regions,^18,19^ and this has been exploited to develop mutants of IgG subclass 1 (IgG1) with enhanced oligomerization potential that are able to activate complement more effectively than wild-type IgG.^20,21^ This was achieved by forming hexameric Fc-platforms, to which the C1 complex binds. C1q is a disulfide-linked complex with six flexible arms that bind to the hexameric Fc platform. There is therefore a symmetry match between the Ab-binding C1q complex and the Fc platform. However, structural data have shown that not all C1q arms bind to the hexameric Ab complex,^18,22^ with four or five arms frequently bound instead of all six (Figure 1b). Furthermore, IgM exists preferentially as a pentamer,^11,23^ but both pentameric and hexameric IgM can bind and activate C1.^7^ These observations reveal a symmetry mismatch between C1 binding and the activating Ab complex, which has not been adequately explained.

Formation of these hexameric platforms is reliant upon the ability of the IgG monomers to bind to antigens at a spacing that provides the means to then form Fc-mediated complexes. This in turn depends on the antigen concentration and proximity, as well as membrane fluidity, epitope location, and IgG subclass. These variables have made it difficult to directly determine the effect of Ab valency on complement activation (Figure 1c).

To explore how Ab binding influences complement activation, we use DNA nanotechnology to control antigen valency (Figure 1d). DNA can be designed to self-assemble into nanoscale shapes that are chemically accessible, which allows for nanometer control over the spacing of functional groups.^24−26^ DNA nanostructures have been previously used to template antigens to determine the effect that spacing and valency have on bivalent Ab binding efficiency with respect to Ab affinities.^27^ However, it has not been used to induce, measure, or control complement activation.

Here, we use DNA nanostructures to pattern antigens with defined valencies and distances that enable us to template Ab complexes and assess the effect of geometry on complement activation (Figure 1d,e). Biophysical characterization of Ab binding and complement initiation revealed that preformed Ab complexes activated complement to a greater extent than the same number of Abs left unpatterned, but this effect was dependent on the interantigen distance. Applying these to liposomal cell mimetics allowed us to assess the degree of membrane rupture caused by complement-induced MAC pore formation. Together, these data provide insights into the role of geometry on Ab-mediated complement activation. Furthermore, we augmented these biophysical assays with cryo-electron tomography visualization of DNA nanostructure-mediated complement activation on lipid bilayers in nonpurified human serum. Together, these data provide insights into how the C1 complex is activated by nanopatterned antigen-Ab complexes and suggest routes to modulate complement activation by Ab engineering. Furthermore, this research highlights several important caveats that must be taken into consideration for biofunctional DNA nanostructure design before they can be used to modulate extracellular immune system pathways.

## Results

### DNA Nanostructure Design and Characterization

**Figure 2:**
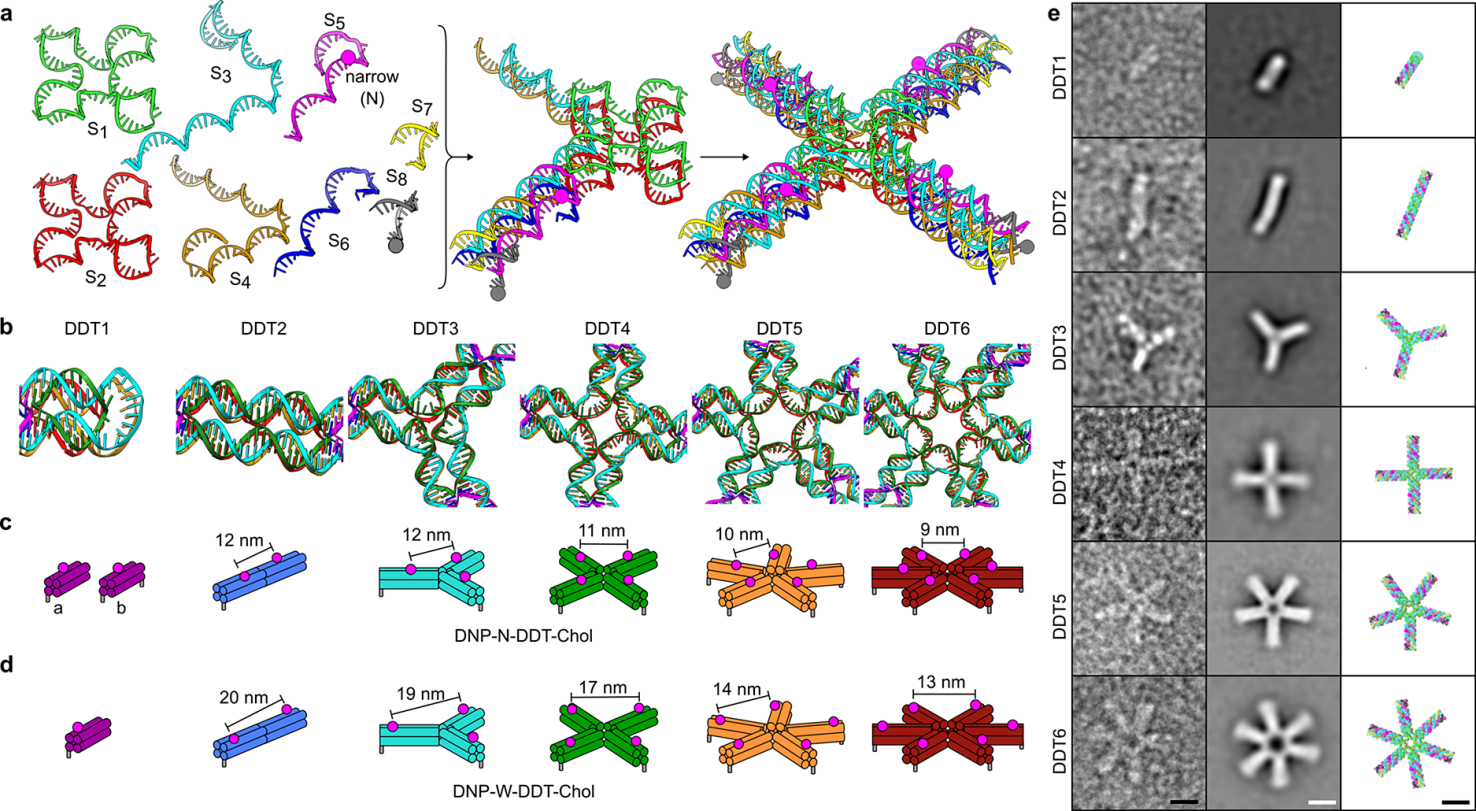
Characterization of DNA nanostructures DDT1–6. (a) Model of strands S_1–8_ forming DDT4. One of each core strand (S_1_ and S_2_) combined with four copies of staple strands S_3–8_. The narrow antigen positioning on S_5_ is indicated by purple spheres, and the cholesterol location on S_8_ is indicated by gray spheres. (b) Detail of modifications to the hub regions necessary to produce DDT nanostructures with defined numbers of arms. (c) Models of DDT1–6, with interantigen distances of narrow positioned antigen 2,4-dinitrophenyl (DNP) shown. Two versions of DDT1 are shown; DDT1_a_ and DDT1_b_ contain one or cholesterol molecules, respectively. (d) Models of DDT1–6, with interantigen distances of wide positioned DNP. (e) EM micrographs, class averages, and models (left–right) of DDT1–6. Scale bars represent 10 nm.

DNA nanostructures were designed to display antigens at specific locations. We used the double-decker tile (DDT) lattice design from Majumder et al. as inspiration,^28^ which comprises rigid arms formed from four interconnected double-stranded DNA helices placed on a 2 × 2 grid held together at a central hub (Figures 2a and S1a). These arms are symmetric, which helps increase the folding yield.^29^ The DDT design is low-profile, with only ∼4 nm height contributed by the two stacked DNA double helices, and the arms are relatively flexible in-plane, which may aid the formation of Ab complexes. The sticky ends of each arm, present in the lattice-forming DDT design,^28^ were either removed (strands labeled “blunt”) or sticky ends were exchanged to thymine bases (Ts; stands labeled “extra-Ts”) to inhibit lattice formation (Table S1).^28^The central hub of the design was modified to generate individual tiles containing one (DDT1), two (DDT2), three (DDT3), four (DDT4), five (DDT5), or six (DDT6) arms, each composed of eight different single-stranded DNA (ssDNA) strands (S_1–8_; Figure 2a,b and Table S1), with two “core” strands that template the number of arms (S_1_ and S_2_) and six “staple” strands, which form the double-decker arms (S_3–8_).^28^ Hinges connecting each arm to a central hub comprised various lengths of unpaired T bases at the bending point of the core strands S_1_ and S_2_, which were specific for each design and required by the DNA geometry.^30^ For DDT1 and DDT2 (no bend), no extra Ts were added, whereas three, four, five, and seven Ts were added for DDT3 (120° bend), DDT4 (90° bend), DDT5 (72° bend), and DDT6 (60° bend), respectively. To generate DDT1, strands S_3_ and S_4_ were redesigned to form intra-arm base pairs, instead of mediating interarm binding, which required the addition of nine Ts to these strands to allow for the 180° bend required (Figure 2b and Table S1).

Antigens comprising the hapten 2,4-dinitrophenyl (DNP), which are tightly bound by anti-DNP Abs (∼0.8 nM K_D_),^31^ were incorporated into the S_5_ strand. This strand is present and sequence-invariant in all DDT designs, and antigens were positioned at discrete locations that were oriented on the upper face of the DDT (Figure S1a,b). One location, named “narrow” (N), was chosen to emulate the distances between antigens determined by structural data,^22,27^ while a second distance, named “wide” (W), was chosen to determine the effect that more widely separated antigens would have on complement activation. These nanotemplates are known as DNP-N-DDT1–6 (for the narrow design) and DNP-W-DDT1–6 (for the wide design) henceforth. The antigen was placed either 6 nm (N) or 10 nm (W) along each DDT arm, as measured from the middle of the nanostructure, yielding antigen–antigen distances of 12, 12, 11, 10, and 9 nm for DNP-N-DDT2, DDT3, DDT4, DDT5, and DDT6, respectively, and 20, 19, 17, 14, and 13 nm for DNP-W-DDT2, DDT3, DDT4, DDT5, and DDT6, respectively (Figure 2c,d). To activate complement, we used human anti-DNP IgG1 Abs, which have been shown to work before.^12,18,20,22^

To bind the DDT1–6 to liposomes, the S_8_ strand on the bottom side of the tiles was modified during synthesis to display a 3′ cholesterol group on a tetra-ethylene glycol (TEG) linker (Figure S1a,c). This cholesterol group was included on the end of a TT-tetra-ethylene glycol (TEG) linker that projects out from the arms and was designed to also serve as a steric hindrance to inhibit intertile π–π stacking and oligomerization.^32−34^ We designed two versions of DDT1 (DDT1_a_ and DDT1_b_), which contained one or two cholesterol molecules, respectively (Figure 2c). DNA nanostructures containing cholesterol are labeled DDT1–6-Chol. A positive control comprised a bifunctional ssDNA strand modified with both DNP and cholesterol (Figure S1d and Table S1). This bifunctional strand was synthesized with a 3′ cholesterol group and a 5′ amine group, which was chemically conjugated to a DNP moiety (see the Supporting Information for details).

DNA nanostructures were folded using thermal annealing, which was monitored using agarose gel electrophoresis and size exclusion chromatography (SEC) (Figure S1e,f). The gel revealed the presence of clear bands corresponding to the folded structures, with minimal unpaired strands at lower molecular weights. Due to the small size of the antigen compared to the overall size of the DNA nanostructure, there was no apparent shift in gel mobility in the presence of DNP. However, the increasing number of arms in each design resulted in clear shifts in both agarose gel mobility and SEC retention volumes, with the main peak of DDT6 eluting at ∼0.95 mL and DDT1 eluting at ∼1.25 mL, with the remaining designs eluting in between in the expected order. SEC also confirmed the high purity of the folded nanostructures, with a low proportion of unpaired strands eluting at higher retention volumes (∼1.73 mL; Figure S1f). Consequently, we used the folded DDT nanostructures without further purification. Next, the correct folding of the DDT designs was assessed using negative stain transmission electron microscopy (TEM). DDT1–6 were readily identifiable in both electron micrographs and class averages (Figures 2e and S2a). These revealed discrete nanostructures with the correct number of arms, and class averages show the limited in-plane flexibility of the rigid arms (Figures 2e and S2a,b). Although the DDT design was originally generated to form extended lattices via sticky-end polymerization (Figure S2c),^28^ by removing the sticky ends, we confirmed that the lattice no longer formed (Figure S2d). Furthermore, to ensure that these blunt ends do not lead to π–π stacking and nonspecific oligomerization of tiles, we also generated versions with four additional Ts at the end of the arm (strands labeled “blunt” or “extra T”, respectively; Table S1).

### Binding Antibodies to DNA Nanostructures

**Figure 3:**
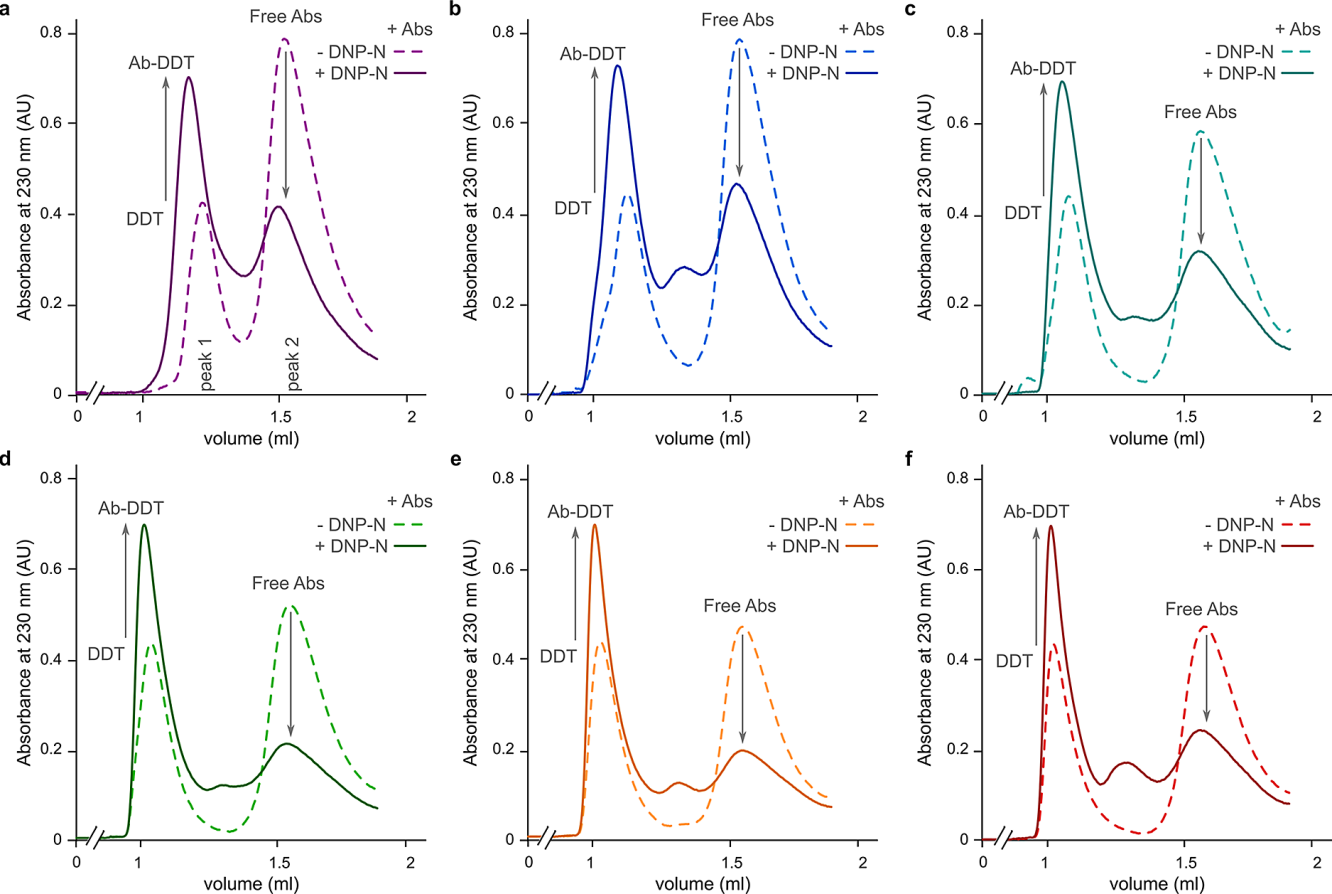
Size exclusion chromatography profiles of DDT nanostructures bound to Abs. (a–f) Abs added to DDT nanostructures ± DNP-N antigens for DDT1 (a), DDT2 (b), DDT3 (c), DDT4 (d), DDT5 (e), or DDT6 (f). Arrows indicate changes in peak intensities upon Ab binding.

The DDT nanostructures were designed to be used as platforms to bind Abs with distinct valencies and distances. Monoclonal anti-DNP IgG1 Abs were incubated with DDT1–6 with and without conjugated DNP at either the narrow or wide position. Agarose gel electrophoresis showed the appearance of bands and smears with lower motility only in the presence of DNP (Figure S3a), indicating Ab binding to DNP-DDTs. To improve our understanding of Ab binding, we performed SEC on DDT nanostructures ±DNP-N and ±Abs, which revealed Ab binding only in the presence of all required components (Figures 3 and S3b). SEC profiles monitored at 230 nm revealed two prominent peaks, labeled 1 and 2 (Figure 3a). In the absence of DNP antigens, peak 1 corresponds to DNA nanostructures (DDT), and peak 2 corresponds to free Abs. In the presence of DNP-N conjugated to the DNA nanostructures, the intensity of peak 1 increases, while that of peak 2 decreases, indicating binding of Abs to DNP-N-DDT designs (Ab-DDT; Figure 3), and a concomitant reduction in free Abs.

### Prepatterning Antibody Complexes using DNA Nanostructures Enhances Complement Initiation

**Figure 4:**
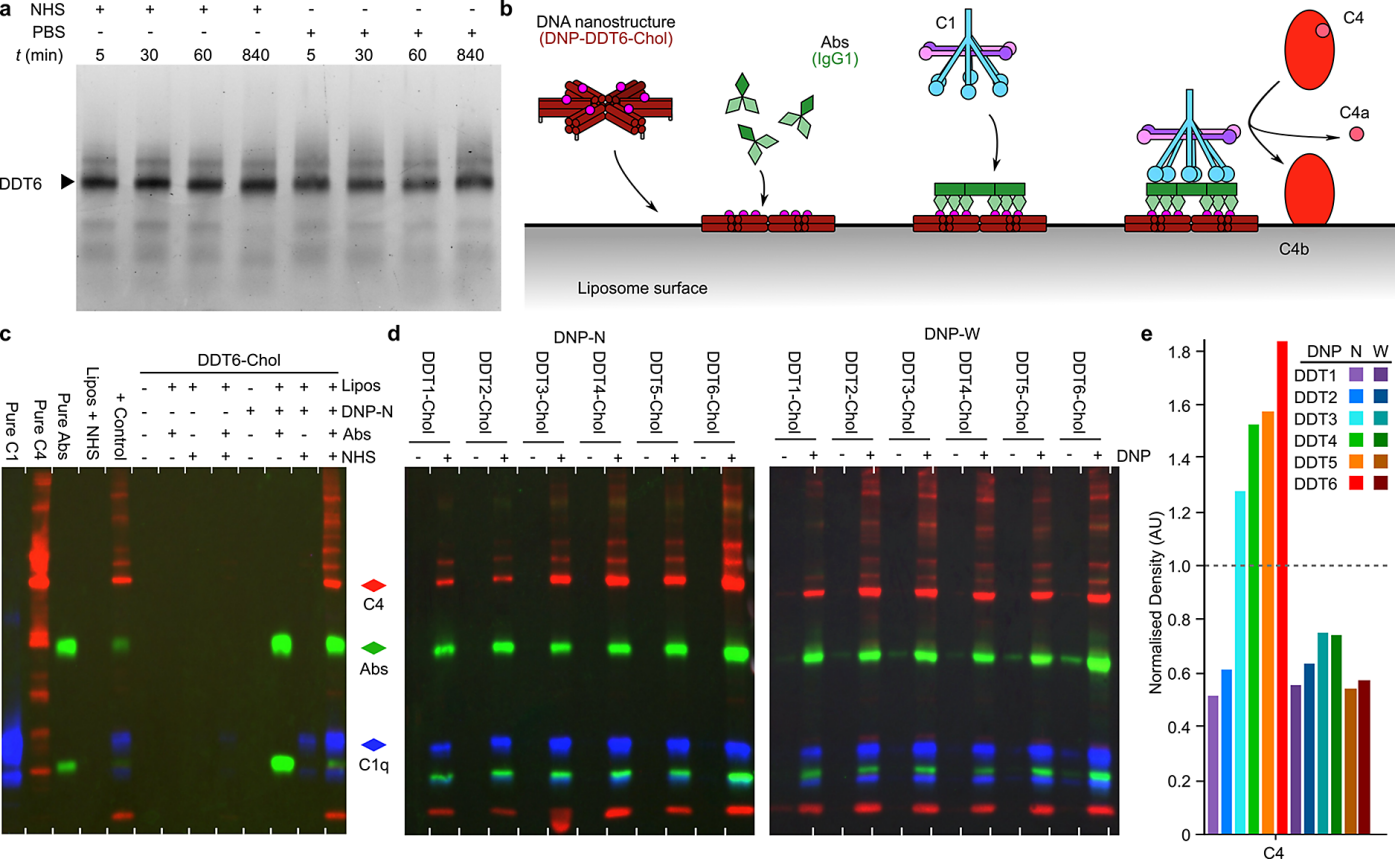
DDT-mediated C1 activation and C4b deposition on liposome membranes. (a) DDT nanostructures are stable for extended time periods in normal human serum (NHS). DDT6 was incubated with NHS or PBS for times (t) of between 5 min and 14 h. (b) Schematic showing the initiating steps of the classical complement cascade mediated via DNA nanostructures. At 4 °C, the complement cascade is limited to C1 activation and C4b deposition. (c) Representative Western blots overlaid showing the detection of C1q (a component of the C1 complex; blue), C4 (labels both C4 and C4b; red), and IgG Abs (green) on liposomes isolated after incubation with the components shown at the top of the gel. Individual detections on the same blot are shown in Figure S4b–d. For the overlaid gels, bands are false-colored for visualization. (d) Overlaid Western blots of C4 copurified with liposomes after complement activation by DNP-N/W-DDT1–6-Chol. IgG Abs (green), C1q (blue), and C4 (red), bands are false-colored during overlaying. Blots were treated the same way as (c) and individual detections can be seen in Figure S5b–d,f–h. (e) Quantitative Western blot analyses using densitometry analysis of the bands indicated by colored arrowheads in (d); Ab values were normalized to 1 before C4 values were compared with respect to the normalized Ab values.

To monitor DNA nanostructure-mediated C1 binding and activation of the C1r_2_s_2_ serine proteases, we followed complement binding and deposition onto liposome surfaces, which act as cell membrane mimetics. Normal human serum (NHS) was used as a source of complement components. To determine the stability of the DDT nanostructures in the presence of NHS, we incubated DDT6 with either phosphate-buffered saline (PBS) or 10% NHS over a period of 14 h (Figure 4a). No difference between the samples in the presence or absence of NHS was apparent, indicating long-term stability of these nanostructures in NHS.

DDT6-Chol ± DNP-N/W (100 nM final DNP concentration; 16.6 nM DDT6 concentration), with an excess of anti-DNP IgG Abs (650 nM final concentration) where indicated, were incubated with liposomes and subsequently cooled to 4 °C. Ice-cold NHS (10% v/v final concentration), which contains all of the relevant complement components, was then added where indicated. At 4 °C, the C1 complex is able to bind to Abs and cleaves C4, but the pathway does not progress beyond C4b deposition (Figure 4b).^7,15^ Liposomes, and any bound material, were then isolated from soluble material by centrifugation.

Silver-stained polyacrylamide gel electrophoresis (PAGE) was used to monitor binding or deposition of proteins and DNA nanostructures onto the liposomes (Figure S4a). Gels showed minimal binding of NHS components to liposomes without a source of antigens present (*Lipos + NHS*). This minimal background binding might be caused by incomplete washing of the liposomes after centrifugation. In contrast, extensive protein binding was observed in the presence of the positive control (+*control*), which comprised a DNA strand modified with both 3′ cholesterol and 5′ DNP. DDT6-Chol was revealed as an isolated band that copurified with the liposomes, indicating successful binding to the lipid membrane. When incubated with Abs, but without DNP antigens, DDT6-Chol showed minimal background binding of serum proteins. Similarly, as long as Abs were omitted, only a negligible amount of protein bound to the liposome in the presence of NHS, again likely caused by incomplete washing, and compared with positive control the bands are faint. In contrast, DNP-N-DDT6-Chol (i.e., DDT6 modified with DNP antigens at the narrow position and containing cholesterol) showed copurification of Abs with the liposomes, indicating DNA-mediated Ab binding to the liposome membrane.

In the presence of DNP-N-DDT6-Chol, Abs, and NHS, extensive protein binding was apparent on the liposome surfaces, indicating successful DNA nanostructure-mediated complement activation. Some proteins were also present in samples without Abs but in the presence of DNP-N-DDT6-Chol and NHS. Here, we also presume that this is due to incomplete washing of the liposomes after centrifugation. To verify that we are observing the predicted proteins bound to our liposomes, we performed Western blotting for the relevant components; IgG Abs, C1q, and C4 (Figure 4c). Each time, the blot was stripped before the detection of the next target (Figure S4b–d). Clear anti-DNP IgG Abs binding to the positive control and DNP-N-DDT6-Chol was visible, confirming that the DNA nanostructure was bound to the liposome and able to bind Abs. A small amount of C1q was detected bound to liposomes without Abs on DDT6-Chol ± DNP-N, but there was no C4 visible, indicating nonactivated C1 presumably associating weakly to the DNP-N-DNA nanostructures or liposomes alone. Overlaying the detection of Abs, C1q, and C4 revealed binding of all three under only two of the conditions; the positive control (+*control*) and the final lane containing DNP-N-DDT6-Chol, Abs, and NHS with liposomes (Figures 4c and S4b–d). This confirms that DDT6 displaying both cholesterol and DNP can activate the complement cascade when bound to the surface of liposomes in the presence of Abs and NHS.

DDT1 is the only design with a single cholesterol moiety binding the nanostructure to the liposome. To determine if the number of cholesterol groups has an effect on DDT-liposome binding, we compared complement activation by DNP-N/W-DDT1_a_-Chol and DNP-DDT1_b_-Chol, which contain one or two cholesterol groups, respectively. Incubating these with Abs and NHS, and again visualizing pulled-down Abs and complement proteins via Western blotting, showed no difference between the presence of one or two cholesterol groups (Figure S4e–h). We also tested whether the addition of extra Ts on the ends of the DDT arms affected binding or complement activation and again observed no difference between the presence or absence of extra Ts (Figure S4e–h), indicating that removal of sticky ends and the extended S_8_ cholesterol-containing strand is sufficient to inhibit nonspecific DDT polymerization.

Next, we determined if C4b deposition on liposomes was dependent on the valency of Ab binding. DDT1–6-Chol ± DNP-N/W were bound to liposomes before incubation with Abs and NHS at 4 °C and purified as described above (Figures 4d and S5). Silver-stained gel electrophoresis showed that, for each DNA nanostructure, proteins bound to the surface of liposomes could be detected if DDTs contain cholesterol and DNP, with minimal background binding to samples without DNP (Figure S5a,e). Using Western blotting to verify that the detected bands were Abs, C1, and C4, we showed copurification of all three components with liposomes for DDT1–6-Chol only in the presence of DNP antigens (Figures 4d and S5b–d,e–h). Densitometry of the bands was used to determine the effect of Abs valency on C4b deposition. Density values were normalized for Ab concentration in order to directly compare the ability of preformed antigens to activate C1 and stimulate the deposition of C4b. This revealed that the amount of C4b deposition was clearly affected by prepatterning the antibodies at the narrow position into complexes, with a clear correlation of increasing C4b deposition with increasing Ab valency (Figure 4e). However, this effect could only be seen using the narrow antigen position. In contrast, positioning the antigens with a higher radius on the nanotemplates removed any preference for preformed geometric patterns, with C4b detection invariant to antigen valency (Figures 4d,e and S5).

### DNA Nanostructure-Templated Antibody Complexes Activate Complement

To enable in situ monitoring of complement activation by DNA nanostructure-mediated Ab complex formation, we bound DDT1–6-Chol to liposome surfaces. Liposomes were formed encapsulating sulforhodamine B (SRB) at a self-quenching concentration.^35^ Complement activation leads to MAC pore formation,^4^ which lyses the liposome and allows dye leakage, resulting in an increase in fluorescence intensity.^7,36^ DNA nanostructures (DDT1–6-Chol) ± DNP-N/W were incubated with liposomes in PBS and thereby functioned as the only antigen source for classical complement activation.

**Figure 5:**
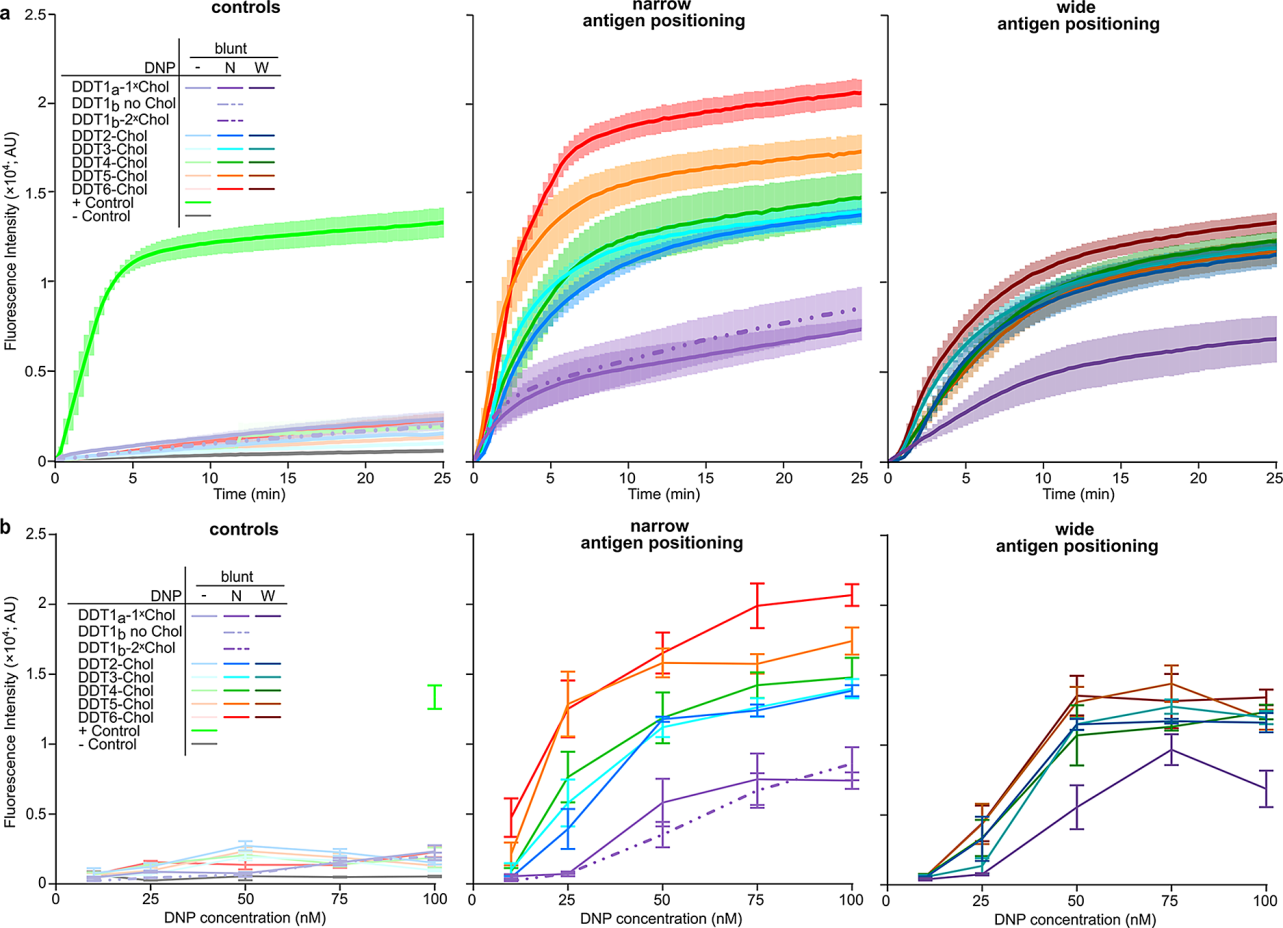
Liposome lysis assays reveal DNA nanostructure-templated Ab complexes can activate complement. (a) Liposome lysis assay showing an increase in fluorescence caused by DNA nanostructure-templated Ab binding at 100 nM DDT. Controls, narrow antigen positioning and wide antigen positioning (left–right) were plotted separately. (b) Fluorescence intensity of DDT1–6-Chol mediated lysis using 10–100 nM DNP, as measured after 25 min. Controls, samples folded with DNP at the narrow position and DNP at the wide position (left–right) were plotted separately. Error bars in all panels represent the standard error of three independent replicates.

Different concentrations of DNP-N/W-DDT1–6, ranging from 10 to 100 nM total DNP (e.g., for 100 nM total DNP concentration, 100, 50, 33.3, 25, 20, and 16.6 nM of DDT1, DDT2, DDT3, DDT4, DDT5, and DDT6, respectively, were used), were incubated with the liposomes for 10 min before an excess of anti-DNP IgG Abs (350 nM final concentration) was added. After a 100 s incubation to allow Abs to bind to the DNA nanostructure-templated DNP antigens, NHS (10% v/v final concentration) was added, and the fluorescence intensity was monitored. No complement activation occurred if no antigen was present, and DNA nanostructures did not lyse the liposomes on their own. In contrast, the positive control, comprising a DNA strand modified with both 3′ cholesterol and 5′ DNP, showed clear complement activation (Figure 5).

At low (10 nM) DNP concentrations, only DNP-N-DDT6 was able to activate the complement system (Figures 5b and S6); no other valency or spacing, including DNP-W-DDT6, displayed any activity. Upon increasing the antigen concentration to 25 nM total DNP, we observed an increase in the fluorescent intensity indicating complement activation mediated by DNA nanostructure-templated Ab complexes with two or more antigens (DNP-N-DDT2–6-Chol) (Figures 5b and 7a). This was greatly reduced for the wide designs, which were consistently worse than the narrow variants. None of the DDT1 constructs activated complement at 25 nM, irrespective of the addition of an extra cholesterol group or extra Ts (Figures 5b and 7a). Importantly, no significant difference between the blunt designs and designs with extra Ts was observed for any construct at any concentration (Figures S6–S10), indicating that the DDT designs are not polymerizing via nonspecific π–π interactions. For each of the concentrations and time points, we calculated p-values using an ordinary one-way ANOVA and visualized these values in a correlation matrix to compare the statistical analyses (Figures S6–S10 and Data S1). These showed that, at 25 nM, only DNP-N-DDT5 and DNP-N-DDT6 were significantly different compared to the same DNA structures without antigens and were also significantly different compared to the widely positioned antigens (Figure S7b). This difference is already apparent after 5 min (Figure S7c,d). Additionally, after 15 min activation, complement activation by DNP-N-DDT4 also becomes significantly higher than the background (Figure S7d).

At 50 nM DNP, the differences between DNP-DDT1-Chol and DNP-DDT2–6-Chol were more apparent (Figures 5b and S8a). Whereas all DNP-N/W-DDT2–6 samples were significantly different compared to samples without antigens (Data S1), none of the DNP-DDT1 samples activated complement to a level significantly higher than background (Figure S8b), which was consistent over all time points assessed (Figure S8c,d). With 75 nM DNP, DNP-N-DDT6-Chol was the most effective complement activator, followed by DNP-N-DDT5-Chol, whereas DNP-W-DDT6 and all other DNP-N/W-DDTs except DDT1 show approximately equal complement activation. However, all DNP-DDT nanostructures are able to activate the complement system at this concentration and are significantly different from the negative controls (Figures 5b and S9). Nevertheless, comparing all DDTs over time with each other, we again observe that DDT1 is less efficient in activating the complement system compared to all other DDTs when antigens are nanopatterned at the narrow position. Again, all DDTs with DNP at the wide position are less efficient than the narrowly positioned antigens (Figure S9c,d).

At the highest used concentration of 100 nM DNP, DNP-N-DDT6 is again the best complement activator, closely followed by DNP-N-DDT5. DNP-N-DDT2–4 displayed comparable complement activation abilities, while DNP-N-DDT1 is again the worst complement activator. However, the wide antigen positioning on the nanostructures leads to less complement activation compared to their narrowly spaced variants. While DNP-W-DDT2–6 were all comparable and better at activating complement than DNP-W-DDT1 (Figures 5 and S10a,b), all were slower to activate the complement system when compared to the narrow versions (Figure S10c,d) and did so to a lesser degree of MAC pore formation. Additionally, the DNP-DDT1 nanostructures were all much slower at activating complement compared to the higher-valency designs (Figure S10c,d).

### Visualization of DNA Nanostructure-Mediated Complement Activation using cryoET

**Figure 6:**
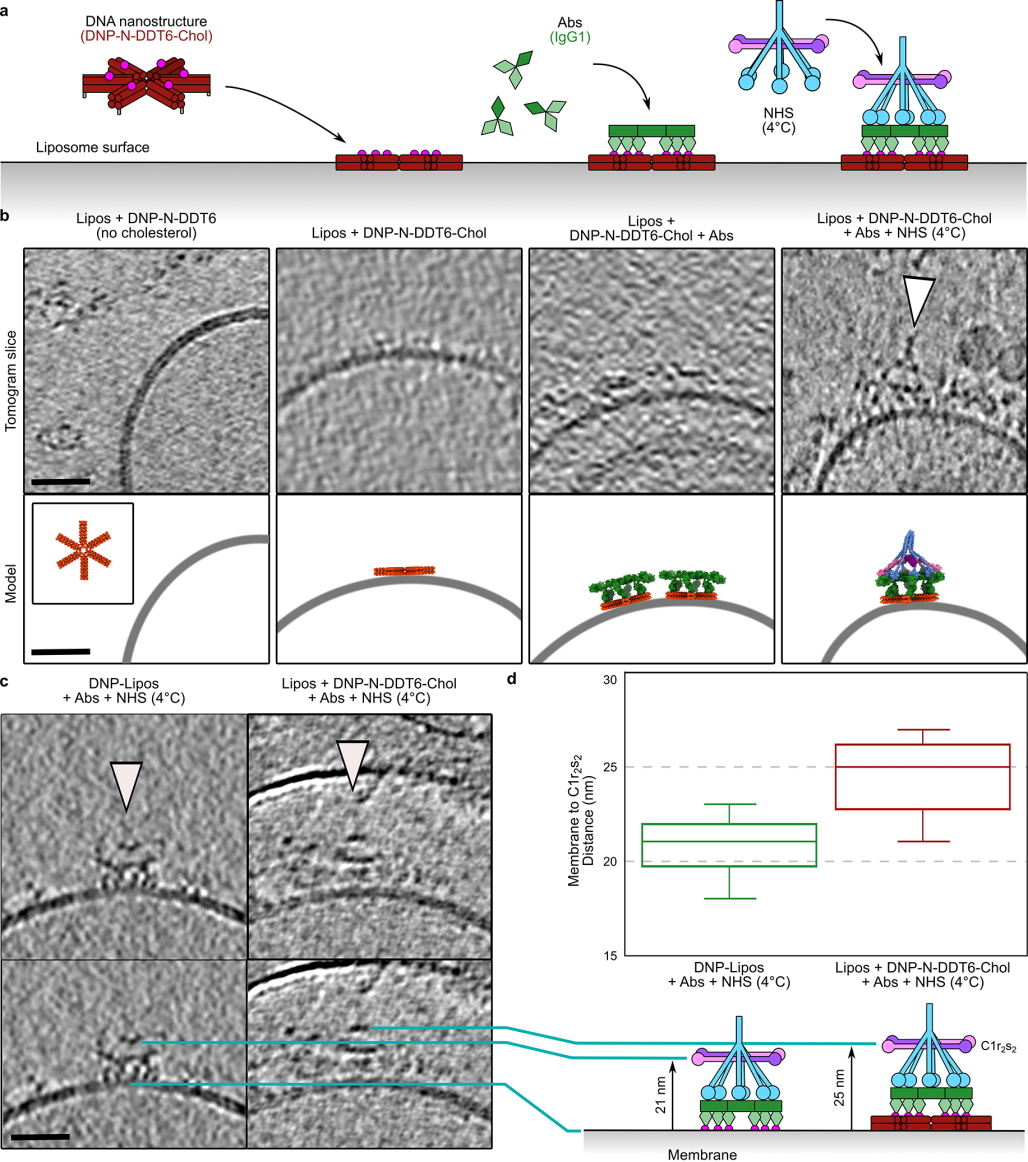
Cryo-electron tomography of DNA nanostructure-mediated complement activation. (a) Schematic of steps required for C1 binding on liposome associated DDT6. (b) Slices, ∼10 nm thick, through cryo-electron tomograms at each step of the schematic shown in (a). (c) Difference in height between C1 bound to Abs on DNP-coated liposomes (left) and DNP-N-DDT6-Chol nanostructures recruited Abs on liposomes. (d) Box plot of height measurements from the liposome membrane to the C1r_2_s_2_ platform visible in the tomogram slices (*n* = 10 for both). The schematics below show how the height was measured. Scale bars all represent 30 nm.

To visualize complement activation by the DNA nanostructure-templated Ab complexes on liposomes, we used cryo-electron tomography (cryoET). Liposomes were incubated with 50 nM DNP-N-DDT6-Chol (Figure 6a). Next, an excess of anti-DNP IgG Abs (final concentration 350 nM) was added and subsequently cooled to 4 °C before the addition of ice-cold NHS (1.5% v/v final concentration), to halt the complement cascade at C4b deposition.^7,15^ Liposome samples were prepared for cryoET that contained (*left to right;*Figure 6a,b); DNP-N-DDT6 ± cholesterol, with anti-DNP IgG Abs, or with all the required components cooled to 4 °C.

In the absence of cholesterol, no DNP-N-DDT6 was observed bound to the liposome membranes, although material with the correct dimensions for solution-phase DNP-N-DDT6 was visible in the vitrified medium (Figure 6b). However, DNP-N-DDT6-Chol was observed on the liposome surfaces visible as low-profile density. Upon the addition of anti-DNP IgG Abs to liposomes with DNP-N-DDT6-Chol, clear platforms appeared (Figures 6b and S11a) that corresponded closely with IgG platforms previously observed using cryoET.^22,37^ Finally, after incubation with chilled NHS, C1 complexes were clearly visible on top of Abs platforms recruited by DNA nanostructure-templated Ab complexes (Figures 6b and S11b). Interestingly, these C1 complexes appeared farther from the membrane than the IgG-C1 complexes previously visualized when bound to antigenic membranes (Figures 6c and S11c).^22^ On liposomes binding anti-DNP IgG Abs directly, the DNP antigens were linked to the lipid. Measuring the distance from the lipid membrane to the C1r_2_s_2_ protease platform, clearly visible in tomograms as a platform parallel to the liposome membrane (Figure 6c,d), yielded different values; C1 bound to Abs on the surface of DNP displaying liposomes and C1 bound to Abs recruited by DNP-N-DDT6-Chol on the surface of liposomes yielded values of 21 and 25 nm, respectively (Figure 6d). Consequently, when bound to DNA nanostructure-templated Ab complexes, C1 was 4 nm farther from the membrane, approximately the same height that two stacked DNA double helices would add.

## Discussion

Antibody engineering has led to the development of monomeric IgG Abs with an enhanced propensity to oligomerize.^18^ Oligomerization of antibodies utilized increased avidity to overcome the weak affinity of C1 for individual Fc domains.^12^ The design and development of these supramolecular Ab complexes required extensive rational and random mutagenesis,^18,20^ which led to a single hexameric species held together via noncovalent interactions between the Fc domains.^19^ This so-called Hexabody technology is being developed as a potential therapeutic to enhance immune responses, such as activation of the complement system.^20^ However, other oligomeric states exist with a multitude of geometric nanopatterns, and it is not yet clear how many of these are sampled by antibodies, or indeed capable of activating the complement system (Figure 1). By using differential combinations of mutations, Strasser et al. could modulate IgG-mediated complement activation based on the oligomeric state.^12^ These data indicated that a minimum of four C1q binding sites were required for complement activation, in contrast to previous data, which showed that two Abs are sufficient.^15−17^ This difference may be due to the extensive mutations required to induce the protein oligomeric state.^12^

**Figure 7:**
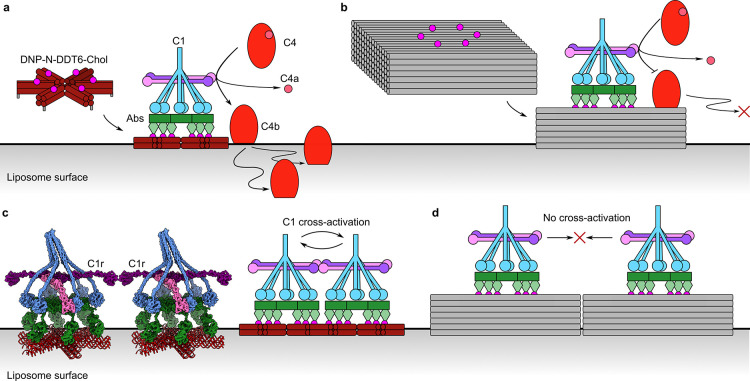
Design considerations for DNA-mediated complement activation. (a) DDT nanostructures are designed to be low-profile, allowing for efficient C4b deposition by active C1 complexes. (b) Larger DNA nanotemplates would introduce steric hindrances to C4b deposition, limiting C4b binding and opsonization. (c) Atomistic models of DDT6-IgG1-C1 complexes cross-activating. Also shown is a schematic representation of C1 cross-activation. (d) Larger DNA constructs would not allow adjacent C1 complexes to physically interact.

By using DNA as a nanopatterning scaffold, we were able to template wild-type (nonmutated) IgG Abs on DNA nanostructures with distinct valencies and geometries and then scan this geometric space with fewer structural perturbations than engineering Abs requires. Our DNA nanostructure design was chosen to be flexible and low-profile to minimize any structural perturbations besides the antigen valency and spacing. Previously, antigens have been nanopatterned on rigid DNA nanostructures,^27^ but these, even when placed on an “idealized” hexameric array, were not shown to activate complement. Active C1 cleaves C4 and deposits C4b on the surface by forming a covalent bond between a reactive thioester within C4b and nearby hydroxyl or primary amine groups (Figure 7a).^7,8^ However, DNA possesses neither of these functional groups and therefore may not be a suitable surface for C4b deposition (Figure 7b). Multiple copies of C4b are deposited and opsonize the target surface,^4,5^ and deposited C4b binds to C2 before cleavage by C1s to form the C4b2b C3 convertase enzyme. This means that C4b must be able to diffuse away from the C1 complex to both function as an opsonin and to allow binding of C2 before cleavage. With these considerations, we designed the DDTs to be as small as possible, such that any steric hindrance for C4b deposition was minimized (Figure 7a). Larger DNA constructs, such as those used in Shaw et al.,^27^ could inhibit C4b deposition (Figure 7b),^7,37^ leading to stalled complement progression and no MAC pore formation. Furthermore, the distance of the C1 complex from the surface impacts complement activation, with taller constructs less able to activate complement.^38^

Complement activation by the C1 complex is induced upon C1q binding, which leads to the activation of C1r within the heterotetrametric C1r_2_s_2_ platform. It is not yet known if C1r activation proceeds via autoactivation (i.e., C1r cleaves the adjacent C1r within the same C1r_2_s_2_ platform), or cross-activation (C1r cleaves the C1r of an adjacent C1 complex). By minimizing the size of our DNA nanotemplates and designing flexible arms instead of a rigid sheet, we have avoided streric inhibition of cross-activation, and allowed either autoactivation or cross-activation to occur (Figure 7c). Again, larger DNA constructs could inhibit C1 cross-activation (Figure 7d), thereby limiting complement activation. Indeed, our DNA nanostructures are able to diffuse along the lipid membrane, and this allows for lateral C1 interactions that have been previously posited to be present during C1 activation.^22^ Although our DNA structures are small enough to enable C1 cross-activation, we attempted to limit artificial cross-activation induced by the DDT nanostructures polymerizing via nonspecific π–π stacking between blunt-ended DNA bases by including an extension on strand S_8_, but we also assessed this by comparing DDTs with and without additional Ts at the end of each DDT arm. The addition of Ts did not lead to a difference in complement activation compared to blunt-ended tiles (Figures S6–10).^7,37^

Clearly, the impact of the shape and chemistry of DNA nanostructures are important parameters to take into consideration when designing DNA nanotemplates. For use in biological or biophysical assays, the scalability and variability of the DNA design should also be taken into account. The DDTs utilized in this study were designed such that the modifications required to change the antigen valency were minimal. Unlike nanostructures based on DNA origami, DDTs are composed of eight small ssDNA oligonucleotides and do not require a ssDNA scaffold. They are also based on a symmetric design,^28,29^ which greatly reduces the number of strands required. Furthermore, the DNP antigen was incorporated at different positions during oligo synthesis and this strand was present in all DDT designs. This has two benefits: the financial outlay is reduced, as most of the same strands can be used for each design; and there are minimal differences between the designs (only two of the eight strands were altered for each design; Table S1), which allowed for more robust comparisons between their complement-activating abilities.

Regarding DNA-mediated complement activation, DDT1 was the worst construct for both C4b deposition (Figure 4) and MAC pore formation (Figure 5), presumably due to the requirement of IgG Abs to bind to each DDT1 individually and then rely on lateral diffusion to form higher order Ab oligomers that are capable of C1 binding and activation.^12^ We did not observe any difference between DNP-DDT1 constructs, with one or two cholesterol moieties producing equivalent complement activation (Figures 5b and S6–S10).

Comparing the DDT designs displaying the antigens at the narrow position revealed that C4b deposition is proportional to the number of antigenic arms within the DDT designs (Figure 4), and MAC pore formation increased concurrently with the number of antigenic arms present within the DDT designs (Figure 5). DNP-N-DDT6 was the only DNA structure that was able to activate the complement system at a concentration of 10 nM (Figure S6), while the other constructs required 25 or 50 nM antigen to lyse liposomes (Figure 5b). In contrast, DDT1 needed at least 75 nM DNP to achieve significant lysis. For the narrow antigen radius, although C4b deposition improved with higher valency (Figure 4e), this trend was reduced at the stage of liposome lysis via MAC pore formation, with significant differences between DDT1 and DDT2–6 starting at 50 nM DNP and above (Figures 5 and S6–10). This discrepancy may be caused by a reduced ability of C1 to activate on nanopatterns with lower valency, but still produce sufficient C4b to initiate the complement cascade. This proteolytic cascade results in a positive feedback loop that may mask the differences in C4b deposition by amplifying the cascade such that equivalent numbers of MAC pores are formed, leading to equivalent liposome lysis. Interestingly, DNP-N-DDT2–4 were broadly similar in their membrane lysing abilities (Figure 5b), indicating that two proximal antigens are indeed sufficient to display Abs in a conformation able to activate complement, which is in agreement with previous data.^7,15−18,22,27,37^

Why two antigens are sufficient to activate the classical complement pathway and lyse liposomes is not yet clear. We cannot exclude the possibility that two antibody-bound DNA nanostructures are able to align and enable C1 binding. Indeed, this possibility was included in the design to allow for cross-activation of the C1 complexes, as described above (Figure 7). C1 binding to multiple DNA nanostructure-templated Abs complexes is presumably how DNP-DDT1-Chol is able to significantly activate complement above 75 nM concentration. However, below that concentration, DDT1 is not able to activate complement, indicating that at this concentration, the other designs are also sufficiently separated such that only one C1 complex binds per DNA nanostructure. DNP-N-DDT6 was superior to DNP-N-DDT5 at liposome lysis, with DDT2–4 achieving similar levels of lysis. Most likely, DNP-N-DDT6-templated hexameric antibody platforms can bind all six arms of the C1q complex to achieve maximal C1 activation. Previous models of C1 activation have utilized either a “compaction” of the C1q arms^22^ or a sliding motion within the C1r_2_s_2_ platform upon C1q binding to antibodies.^7^ To understand the structural biology of DNA nanostructure-mediated complement activation, we attempted to image the samples using cryo-electron tomography (Figure 6). Tomographic volumes revealed the C1 complexes binding to DNA nanostructure-templated antibody platforms, although we were unable to resolve a 3D map of the entire complex. We posit that this was due to the flexibility of the system; previous maps have been hampered by flexibility and therefore only partially resolved,^22^ and we have added a layer of complexity with the addition of flexible DNA nanostructures. Nevertheless, we were able to image stepwise reconstitution of liposome-bound DDT6-Chol, Abs binding to DNP-N-DDT6-Chol, and C1 complex association to the DNA nanostructure-templated Ab complexes (Figure 6).

While the narrowly spaced antigens revealed a correspondence between the valency and complement activation, this effect was removed if the antigens were positioned at a wider radius, with 2–6 antigens all performing equally as well at membrane lysis and better than one antigen (Figures 4e and 5b), but consistently worse than the narrowly spaced antigens. This is likely due to Ab oligomerization; closely spaced antigens are able to bind Abs such that they can form Fc platforms via supramolecular interactions, but widely spaced antigens limit Fc oligomerization by physically separating the monomeric Abs and inhibiting inter-Fc interactions. These inter-Fc interactions, although relatively weak (∼17.5 mM *K*_D_),^19^ are known to be important for C1 activation,^18^ and enhancing Fc oligomerization by strengthening these interfaces leads to stronger complement activation.^20^ Geometrically limiting or enhancing these interactions by DNA-mediated Ab nanopatterning is therefore a route to control the strength of complement activation.

Our work represents the first time that DNA nanostructure-templated antibodies have been used to activate the complement system. By varying the valency of the nanotemplates, we could control the geometric parameters of complement activation. Recent work has shown that higher Fc platforms behave differently,^37^ with taller IgG3 complexes better able to activate complement than shorter IgG. The work described herein shows a route to explore this further, by systematically varying the height of the initiating complex via the use of DNA templates of a defined height. This may lead to a greater understanding of how the epitope location relates to complement activation.^21,39,40^

We also discovered a correlation between antibody valency and C4b deposition, which implies a potential mechanism to switch complement from an inflammatory response to silent clearance. Although inflammation is generally desired to fight infections, silent clearance of cellular debris is optimal to limit sensitization of the immune system toward benign cellular material, which therefore limits autoimmune responses.^41−43^ By controlling C4b deposition in vivo, it may be possible to limit opsonization to induce phagocytosis without the formation of MAC pores. DNA nanostructures can be targeted to distinct cell types via binding motifs such as aptamers^44^ or directly displaying ligands for receptor binding^45^ and are stable in serum and in vivo (Figure 4).^46,47^ The use of DDT2–4 to induce in vivo dimerization, trimerization, or tetramerization of IgG, instead of hexamerization of Abs, would be a novel route to induce sublytic complement activation.

## Supporting Information Available

The Supplementary data contain the raw and analyzed data sets from all burst assays (Excel tables and GraphPad file). The Supporting Information is available free of charge at https://pubs.acs.org/doi/10.1021/jacs.4c02772.

One-way ANOVA (ZIP)
Experimental section including the design and production of DNA nanostructures, antibody production, liposome production, biochemical assays, and microscopy information (PDF)
